# Antibody-drug conjugates and predictive biomarkers in advanced urothelial carcinoma

**DOI:** 10.3389/fonc.2022.1069356

**Published:** 2023-01-04

**Authors:** Sarah E. Fenton, David J. VanderWeele

**Affiliations:** ^1^ Robert H. Lurie Comprehensive Cancer Center, Northwestern University, Chicago, IL, United States; ^2^ Department of Medicine, Feinberg School of Medicine, Northwestern University, Chicago, IL, United States

**Keywords:** biomarker, antibody-drug conjugate (ADC), urothelial carcinoma (UC), predictive, bladder cancer

## Abstract

The use of antibody-drug conjugates (ADCs) is expanding in several malignancies, including urothelial carcinoma where two of these medications have been approved for use and several others remain under study. ADCs act by binding to specific cell surface proteins, delivering anticancer agents directly to the target cells. Preclinical studies suggest that loss of these surface proteins alters sensitivity to therapy and expression of target proteins vary significantly based on the tumor subtype, prior therapies and other characteristics. However, use of biomarkers to predict treatment response have not been regularly included in clinical trials and clinician practice. In this review we summarize what is known about potential predictive biomarkers for ADCs in UC and discuss potential areas where use of biomarkers may improve patient care.

## 1 Introduction

Urothelial carcinoma (UC) of the bladder is the fourth most frequent cancer of male adults in the United States, with approximately 81,200 cases expected in 2022 (1). Outcomes are driven by the stage at which the disease is diagnosed, with a 5-year overall survival (OS) rate greater than 90% for patients diagnosed with non-muscle invasive bladder cancer (NMIBC), 50-82% for muscle invasive bladder cancer (MIBC) and 5% for metastatic disease (2). Currently approved management strategies for metastatic disease include chemotherapy such as GC (gemcitabine and cisplatin or carboplatin) or MVAC (methotrexate, vinblastine, doxorubicin, and cisplatin), immunotherapy (pembrolizumab, atezolizumab, or avelumab), or targeted therapy (erdafitinib for patients with certain *FGFR3* or *FGFR2* mutations). These therapies are limited by the toxicities associated with active chemotherapy regimens, the low response rates seen with immunotherapy, and the limited number of patients harboring the relevant biomarker. Fortunately, several novel therapies have been introduced in the management of advanced and metastatic disease that have the potential to improve these outcomes. Specifically, the introduction of antibody-drug conjugates (ADCs) such as enfortumab vedotin (EV) and sacituzumab govitecan (SG) have expanded treatment options for patients with advanced UC. Predictive biomarkers that could help to forecast outcomes following the use of specific therapies have not been incorporated into clinical practice. As treatment options are added to the clinician’s armamentarium, biomarkers become valuable in prioritizing active therapies for individual patients. For example, both EV and SG are approved as third line therapies for metastatic UC. Biomarker-driven data may aid in therapy selection between these two agents. Data from preclinical models and secondary analyses of clinical trials using ADCs suggest that several biomarkers exist that may improve therapy selection. Thus, in this review we will cover currently available information supporting the potential use of biomarkers in the management of advanced UC.

## 2 Antibody drug conjugates

ADCs were first synthesized in the 1960s when small molecule chemotherapies were linked to immune gamma globulins (3). Since then, nine ADCs have been approved by the FDA for use in cancers including UC, lymphoma, acute myelogenous leukemia, and breast cancer (4, 5). ADCs are composed of a monoclonal antibody that binds to specific target antigens on the surface of tumor cells linked to anticancer agents such as chemotherapy drugs (6). By delivering these anticancer agents directly to the target cells, ADCs increase therapeutic efficacy while decreasing potential systemic toxicities (7). Many of the antibodies used in the synthesis of ADCs are composed of immunoglobulin G and, instead of optimizing their ability to elicit an immune response, these antibodies are selected based on binding affinity, potential cross-reactivity, and immunogenicity (7, 8). Activation of immune responses following exposure to ADCs can lead to the formation of additional antibodies that bind to the ADC and compromise its efficacy (9, 10). Thus, due to its long half-life, stability in the serum and wide distribution in the intra and extravascular compartments, immunoglobulin G is an ideal candidate in the construction of ADCs (8). The ratio between the drug and the antibody must also be addressed, as a high ratio results in aggregation and clearance of the ADC while a low ratio results in low efficacy following drug delivery (11). Finally, the linker between the drug and the antibody must be optimized to allow stability in the circulation. Early release in the serum can lead to undue toxicity and decreased effect at the tumor (12, 13). However, in most cases the linker must also be able to be cleaved once the ADC is internalized into the target cell (14). Despite these requirements, the introduction of ADCs into clinical practice has been increasing significantly over the past decade. **Table 1** summarizes ADCs that have been studied in UC and their mechanism of action. **Table 2** summarizes ongoing studies investigating the role of ADCs in UC. Unfortunately, many of these ADCs have been studied independent of biomarker testing to predict treatment response, toxicities and potential resistance mechanisms.

**Table 1:** Currently approved ADCs in the management of UC.

ADCs Studied in UC	Antibody Target	Mechanism of Action
Enfortumab vedotin	Nectin-4	monomethyl auristatin E (MMAE) is delivered to the tumor cell, resulting in microtubule inhibition, disruption of cell division and cell death
Sacituzumab govitecan	Trop-2	SN-38 is delivered to the tumor cell, resulting in inhibition of topoisomerase I and the accumulation of lethal DNA double strand breaks
Sirtratumab vedotin	SLITRK6	MMAE is delivered to the tumor cell, resulting in microtubule inhibition, disruption of cell division and cell death
Trastuzumab emtansine	HER-2	Emtansine is delivered to the tumor cell, resulting in microtubule inhibition, disruption of cell division and cell death
Disitamab vedotin	HER-2	MMAE is delivered to the tumor cell, resulting in microtubule inhibition, disruption of cell division and cell death

**Table 2:** Currently active trials of antibody-drug conjugates in urothelial carcinoma.

Ongoing ADC Trials in UC				
Clinicaltrials.gov Number	Treatment Regimen	Patient Population	Primary Outcome Measure(s)	Current Study Status
NCT04223856	EV & Pembrolizumab	Unresectable or metastatic UC	PFS & OS	Recruiting
NCT04963153	EV & Erdafitinib	Metastatic UC	Incidence of AEs, RP2D & MTD	Recruiting
NCT04878029	EV & Cabozantinib	Unresectable or metastatic UC	RP2D	Recruiting
NCT04960709	EV, Durvalumab & Tremelimumab or Durvalumab & EV prior to cystectomy	Cisplatin ineligible (or refuse) MIBC	pCR, EFS & rates of AEs	Recruiting
NCT04700124	EV & pembrolizumab prior to cystectomy	Cisplatin ineligible MIBC	pCR & EFS	Recruiting
NCT03924895	EV & pembro prior to cystectomy	Cisplatin ineligible (or refuse) MIBC	EFS	Recruiting
NCT05524545	EV & Evorpacept	Unresectable or metastatic UC	DLTs & rate of AEs	Recruiting
NCT04527991	SG	Unresectable or metastatic UC	OS	Recruiting
NCT05581589	SG prior to cystectomy	Non-urothelial MIBC	pCR	Not yet recruiting
NCT05226117	SG prior to cystectomy	MIBC	pCR	Recruiting
NCT03547973	SG combination therapy	Unresectable or metastatic UC	ORR & PFS	Recruiting
NCT04863885	SG, Nivolumab & Ipilimumab	Cisplatin ineligible metastatic UC	MTD & ORR	Recruiting
NCT05327530	SG & avelumab	Unresectable or metastatic UC	PFS & rate of AEs	Recruiting
NCT04724018	SG & EV	Unresectable or metastatic UC	MTD & DLT	Recruiting
NCT02675829	T-DM1	HER-2+ solid tumors	ORR	Recruiting
NCT04073602	Disitamab vedotin	HER-2- unresectable or metastatic UC	ORR	Active, not recruiting
NCT05302284	Disitamab vedotin plus toripalimab	HER-2+ unresectable or metastatic UC	PFS & OS	Recruiting
NCT05016973	Disitamab vedotin & triplizumab followed by cystectomy	MIBC	Pathologic response	Not yet recruiting
NCT04879329	Disitamab vedotin & pembrolizumab	Unresectable or metastatic UC	ORR	Recruiting
NCT05495724	Disitamab vedotin & tislelizumab	HER-2+ high risk MIBC	pCR	Recruiting
NCT04839510	MRG002	Unresectable or metastatic UC	ORR	Recruiting
NCT05488353	Disitamab vedotin & penpulimab prior to cystectomy	Cisplatin-ineligible MIBC	pCR	Recruiting
NCT03523572	Trastuzumab deruxtecan & nivolumab	Advanced breast and UC	DLT and ORR	Recruiting

EV, enfortumab vedotin; UC, urothelial carcinoma; PFS, progression free survival; OS, overall survival; AEs, adverse events; RP2D, recommended phase 2 dose; MTD, maximum tolerated dose; MIBC, muscle invasive bladder cancer; pCR, pathologic complete response; EFS, event free survival; DLT, dose limiting toxicity; SG, Sacituzumab govitecan; ORR, overall response rate; T-DM1, Trastuzumab emtansine.

## 3 Enfortumab vedotin

EV is a fully human monoclonal antibody conjugated by a protease-cleavable linker to monomethyl auristatin E (MMAE). The delivery of MMAE to the tumor cell results in inhibition of microtubule formation and cell death through interruption of cell division (15, 16). The antibody component of EV binds to Nectin-4 on the surface of the cell, an immunoglobulin-like transmembrane protein that participates in the formation of the adherens junction (17–20). Nectin-4 is enriched in placental and embryonic tissues as well as squamous epithelial tissues such as the skin where it plays a role in cellular adhesion and immune evasion (21, 22). Nectin-4 is also overexpressed in several cancers including UC as well as breast, ovarian, lung and gastric carcinomas (14, 17, 23–25). In upper tract UC, upregulation of Nectin-4 is associated with poor outcomes, including a shortened progression free survival (26). By targeting Nectin-4, EV delivers MMAE directly to the tumor cells. Although response to EV has not been proven to correlate with levels of Nectin-4, in preclinical models resistance to EV can be induced through downregulation of Nectin-4 suggesting a relationship between Nectin-4 levels and EV sensitivity (19).

EV was granted accelerated FDA approval in December of 2019 for the treatment of patients with advanced UC that progressed following prior treatment with chemotherapy and immunotherapy. This approval was based on the results of a phase II single arm trial evaluating EV as third line therapy. The overall response rate in this study was 44% (95% confidence interval (CI) 35.1 – 53.2) with a median response duration of 7.6 months (95% CI 6.3 – not reached) (15). EV was fully approved following the completion of the phase III EV-301 study comparing EV to physician’s choice chemotherapy (docetaxel, paclitaxel or vinflunine). Compared to chemotherapy, EV significantly prolonged meaningful outcomes with similar toxicity rates. More specifically, the median progression free survival with EV was 5.55 months versus 3.71 months (HR 0.62; 95% CI 0.51 to 0.75, p<0.01) and the median overall survival was 12.88 months versus 8.97 months (HR 0.7; 95% CI 0.56 – 0.89, p=0.001) (27).

### 3.1 Nectin-4 as a potential biomarker for EV

As previously discussed, preclinical models of EV in UC suggest downregulation of Nectin-4 on tumor cells is associated with resistance to therapy (19). However, Nectin-4 has not been incorporated as a predictive biomarker of treatment response. In the phase I trial of EV in UC, evaluation of Nectin-4 expression by immunohistochemistry (IHC) was initially required for enrollment. For each patient, Nectin-4 expression was quantified using a histochemical scoring system based on staining intensity multiplied by percent of cells with positive stain (H-score, range 0-300). Nectin-4 was detected in 97% of patients with a median H-score of 290 (H-score range 14-300). Due to the consistently high expression of Nectin-4, positive testing was removed from the eligibility criteria (16). Recent data from the EV-103 study of EV plus pembrolizumab in metastatic UC confirmed these findings, where a moderate to strong H-score (H ≥ 100) was identified in 92% of patients. Response to therapy was independent of Nectin-4 expression level (28). However, further testing has suggested that Nectin-4 expression may not be as consistent as previously observed.

A study of over 500 bladder tumors identified moderate to strong H-scores (H ≥ 100) in 60% of patients. Rates of strong staining were lower in metastatic sites than in primary tumors (12% versus 34%) (17). Another case series found similar variability in Nectin-4 staining based on tumor stage. Among the NMIBC cases evaluated 87% were positive for Nectin-4 staining, while rates of positive staining dropped to 68.2% in the muscle invasive cases. Nectin-4 positivity also varied based on disease histology, with decreased positive staining in tumors with squamous differentiation (70%), plasmacytoid variants (62.5%), sarcomatoid (10%) and small cell variants (0%) (29). This heterogeneity of expression was also seen in a Japanese retrospective study of patients with MIBC. Interestingly, Nectin-4 expression did not change significantly in samples obtained from patients at the time of transurethral resection (TUR) versus radical cystectomy (RC) when chemotherapy was not administered. However, for patients that received neoadjuvant chemotherapy there was a significant decrease in Nectin-4 expression between these samples. Additionally, in some patients where tissue from a metastatic site was available Nectin-4 expression was decreased in the metastatic site compared to the primary tumor (30).

Recent work has stratified UC into different molecular subtypes, and these categories are associated with variable response to treatment. For example, the majority of luminal and neuroendocrine subtypes have an improved response to atezolizumab therapy, while luminal nonspecified and basal/squamous respond to cisplatin-based therapies. Evaluation of Nectin-4 expression has also identified heterogeneity between these categories. Nectin-4 expression is highest in luminal subtypes (luminal papillary, luminal nonspecified, luminal unstable) compared to basal, neuroendocrine or stroma-rich subtypes. This difference persists after cisplatin exposure (19). These studies suggest greater heterogeneity in Nectin-4 expression than was previously seen in the phase I trial of EV therapy. However, further work is necessary to determine if expression correlates with disease response to EV therapy, whether expression changes based on prior therapies and biopsy site (primary versus metastatic) and whether use of Nectin-4 as a predictive biomarker improves therapy selection and patient outcomes. Answers to each of these questions will be necessary in determining the utility of Nectin-4 as a predictive biomarker to EV therapy.

### 3.2 TP53 and CDKN2B mutations as potential biomarkers for EV

Previous investigations of potential biomarkers for EV therapy have focused on Nectin-4, as it is considered a critical component of ADC localization and binding to bladder cancer cells. However, an additional retrospective study of 28 UC patients identified further potential predictive biomarkers. Patients that were responders to EV (either had a complete response or remained on EV therapy for over 6 months) were enriched for mutations in *TP53* and had improved outcomes including progression free and overall survival. Patients that were nonresponders to EV therapy were more likely to have metastatic disease to the bone and were enriched for mutations in *CDKN2B* (31). Given the limited number of patients included in this study, the identification of two novel predictive biomarkers suggests that more may be found with additional studies.

## 4 Sacituzumab govitecan

SG is a humanized antibody against Trop-2 that is linked with a hydrolysable linker to the small molecule chemotherapy agent SN-38 (32, 33). An inhibitor of topoisomerase I (topo-I), SN-38 acts to destabilize the interaction between this protein and the DNA resulting in lethal DNA double strand breaks during replication that cannot be repaired (34). Trop-2 is a glycoprotein found on the cellular membrane of trophoblastic cells during development as well as stratified squamous epithelial cells in the skin, esophagus, tonsillar crypts and uterine cervix where it plays a role in calcium signalling, proliferation and several other regulatory signalling pathways (35–38). Trop-2 is also overexpressed in epithelial cancers including up to 83% of UC where it is associated with aggressive disease and poor prognosis (36, 37, 39). SG is currently approved for use in triple negative breast cancer (TNBC) and UC, however continued approval for use in bladder cancer patients will depend on the outcomes observed in the phase III TROPiCS-04 trial (NCT04527991).

### 4.1 Trop-2 as a potential biomarker for SG

An initial exploratory trial of SG in solid tumors found that 64% (n=16/25) of the tumors tested were positive for Trop-2 by IHC, however no correlation was observed between Trop-2 expression and response to SG (40). This finding may have been limited due to tumor heterogeneity or sample size, as further studies in UC have identified a positive correlation between Trop-2 expression and response to SG therapy (41). Preclinical testing in TNBC and UC also suggest that modification of Trop-2 expression alters sensitivity to SG (42, 43). However, Trop-2 staining has not been included in the phase II study of SG in UC (44). Thus, its utility as a predictive biomarker remains limited despite secondary analysis in breast cancer studies suggesting Trop-2 expression levels correlate with response to SG therapy. More specifically, a secondary analysis of the phase III ASCENT trial where patients with previously treated, metastatic TNBC were treated with SG versus physician’s choice of therapy found that treatment with SG resulted in benefit regardless of Trop-2 expression, however the benefit was lower in patients with lower Trop-2 expression levels. Median progression free survival was 6.9 months in patients with high expression of Trop-2 on their tumor, 6.5 months for patients with medium expression and 2.7 months with low Trop-2 expression. Response rates and median overall survival mirrored these trends (45). Another study of Trop-2 expression evaluated 9 tumor samples from healthy donors, 21 NMIBCs and 10 MIBCs. Trop-2 staining was positive in the superficial layer of the bladder urothelium in normal samples and increased in NMIBC biopsies. However, the most significant increase in Trop-2 expression was noted in samples with disease invasion, particularly in the cells invading the muscle (36). Similar to Nectin-4, Trop-2 expression also varied in between molecular categories of UC with the highest expression in basal, luminal and stroma-rich subtypes and low levels of expression in neuroendocrine subtypes. Staining for Trop-2 was highly variable in the basal and stroma rich subtypes. Secondary analysis of tissue obtained in the IMvigor210 trial suggested Trop-2 expression remained comparable across metastatic sites and between patients with locally advanced or metastatic UC (43). Thus, Trop-2 remains a potentially useful predictive biomarker for SG response in UC. However, further studies are necessary to confirm this hypothesis.

### 4.2 Topoisomerase-I as a potential biomarker for SG

SG acts by inhibiting topo-I activity, suggesting expression of this enzyme may be a potential biomarker of treatment response and resistance. Studies suggest between 56 and 63% of UCs overexpress topo-I (34, 46). However, further work is necessary to correlate topo-I with outcomes following SG therapy.

## 5 Sirtratumab vedotin

Previously referred to as ASG15-ME, sirtratumab vedotin (SV) is a humanized IgG2 antibody against SLITRK6 linked by a protease cleavable linker to MMAE. SLITRK6 is a transmembrane protein that is overexpressed in UC, breast and lung cancer as well as glioblastoma multiforme (47, 48). In a phase I trial of 51 patients with metastatic UC, 93% stained positively for SLITRK6 (48). However, evaluation of whether expression of this therapeutic target would serve adequately as a predictive biomarker of treatment response remains lacking.

## 6 HER-2 targeted therapy

Several HER-2 targeted therapies are under investigation in the treatment of UC. Although initial trials with lapatinib and trastuzumab did not show efficacy, more recent trials with ADCs show promise (49–51). Trastuzumab emtansine (T-DM1) is composed of an anti-HER2 antibody joined with a non-cleavable linker to emtansine, a microtubule inhibitor. Preclinical models suggest efficacy in UC cells that overexpress HER2. Although a phase II basket trial showed no response in UC patients, several studies are ongoing (NCT02999672, NCT02675829) (52). A higher response rate of 61% in UC patients was seen in a phase II trial of disitamab vedotin (RC48-ADC), an ADC composed of a humanized anti-HER2 antibody conjugated to MMAE (53, 54). A phase II trial studying MRG002, another ADC composed of a humanized anti-HER2 antibody conjugated to MMAE, has identified a safe treatment dose and has observed an overall response rate of 65% (NCT04839510) (55). Other ADCS that target HER-2 currently under investigation in UC include trastuzumab duocarmazine and trastuzumab deruxtecan. The majority of these trials require HER-2 expression levels that are 3+ by IHC or positive by FISH. However, recent studies of trastuzumab deruxtecan in breast cancer suggest these definitions may be broadened, as responses to this drug were seen in patients with HER2-low tumors (defined as 1+ by ICH or 2+ with negative FISH testing) (56). Studies suggest HER-2 is overexpressed in at most 20% of UCs, but evaluation for HER2-low status has not been investigated (4, 57). Based on these findings, validation of HER-2 levels as a predictive biomarker to aid in therapy selection will be critical.

## 7 Conclusion

In 2021 the American Society of Clinical Oncology released a Report on Progress Against Cancer that underscored the importance of including both tissue and blood-based biomarkers in clinical trials. Clinically useful biomarkers should aid physicians and other health care professionals in predicting response to treatment, potential toxicities and resistance mechanisms to therapy (58). Treatment with ADCs are particularly well suited to the integration of biomarkers, as their efficacy depends on the presence of both the antibody target and sensitivity to the delivered anticancer agent. However, trials that have completed and are currently recruiting patients have by and large forgone this potentially fruitful avenue of investigation. Although initial studies suggested high expression of the ADC target, further retrospective and cohort trials identified heterogeneity in expression of proteins such as Nectin-4 and Trop-2 that appear to be critical for ADC efficacy. These raise questions regarding whether expression levels in the primary tumor can be used to evaluate patients with metastatic disease, as well as whether patients with alternative tissue histologies should undergo further testing prior to therapy initiation. Prior therapeutic exposures may also alter biomarker expression, suggesting repeat biopsies may be necessary. Biomarkers remain an untapped resource in the clinical management of patients with advanced UC, and as Jindal et al. showed in their retrospective study of UC we likely have only uncovered the tip of the iceberg in identifying potentially beneficial biomarkers (31).

## Author contributions

SF and DV contributed equally to the conceptualization, writing, and editing of this article. All authors contributed to the article and approved the submitted version.
